# Vascular Access Management After Kidney Transplantation Position Paper on Behalf of the Vascular Access Society and the European Kidney Transplant Association

**DOI:** 10.3389/ti.2025.14712

**Published:** 2025-11-13

**Authors:** Barış Akin, Tamara K. Jemcov, David Cucchiari, Jan Malik, Gavin J. Pettigrew, Ulrika Hahn Lundström, Gianluigi Zaza, Joris I. Rotmans

**Affiliations:** 1 Department of Surgery, Demiroglu Bilim University Florence Nightingale Hospital, Istanbul, Türkiye; 2 European Kidney Transplant Association, Section of European Society of Transplantation, Amsterdam, Netherlands; 3 Department of Nephrology, Clinical Hospital Center Zemun, Belgrade, Serbia; 4 Faculty of Medicine, University of Belgrade, Belgrade, Serbia; 5 Vascular Access Society, Maastricht, Netherlands; 6 Department of Nephrology and Kidney Transplantation, Hospital Clínic of Barcelona, Barcelona, Spain; 7 Complex Cardiovascular Center, General University Hospital, First Medical Faculty, Charles University, Prague, Czechia; 8 Department of Surgery, University of Cambridge, London, United Kingdom; 9 Department of CLINTEC, Division of Renal Medicine, Karolinska Institutet and Karolinska University Hospital, Stockholm, Sweden; 10 Department of Medical and Surgical Sciences, University of Foggia, Foggia, Italy; 11 Department of Internal Medicine, Leiden University Medical Center, Leiden, Netherlands

**Keywords:** ligation of arteriovenous fistula, kidney transplantation, AVF flow reduction, hemodialysis, kidney failure

## Abstract

There is no consensus on whether to ligate or preserve uncomplicated vascular access (VA) after kidney transplantation (KT), as International Guidelines do not address this issue. Enhanced survival rates of kidney grafts may elevate the risk of cardiac morbidity and mortality due to prolonged exposure to the hemodynamic effects of arterio-venous fistulas (AVF). Although VA ligation reduces left ventricle (LV) mass, its impact on cardiovascular (CV) morbidity or mortality is unclear. High-flow VAs can complicate KT patients, and immunosuppressive medication may increase these complications. Despite preserving VA for future hemodialysis (HD) use, central catheters are used in nearly two-thirds of patients. Detecting transplant patients who can undergo AVF ligation and reconstruction when returning to HD allows for flexible decision-making with a multidisciplinary approach, personally tailored to patients at their discretion. Therefore, an algorithm involving Doppler ultrasound and cardiac evaluation is advisable.

## Introduction

The management of vascular access (VA) after kidney transplantation (KT) is a complex and unresolved issue, particularly in recipients with good allograft function and uncomplicated VA. While preservation is appropriate in cases of poor graft function or need for plasma exchange, the optimal strategy for managing a functioning VA in stable KT recipients is not yet clear. With improved graft survival—averaging 11.7 years for deceased donors and 19.2 years for living donors [1]—clinicians are increasingly confronted with the challenge of balancing future dialysis needs against potential VA-related complications, including cardiovascular morbidity.

Data from over 16,000 patients in the US Renal Data System show that only 40% had an arteriovenous fistula (AVF) in place at the time of hemodialysis (HD) re-initiation after graft failure, while nearly two-thirds started HD with a central venous catheter (CVC), despite efforts to preserve VA [2]. Meanwhile, cardiovascular disease remains the leading cause of death in KT recipients with a functioning graft, with a fivefold higher incidence compared to the general population [3, 4]. AVFs may contribute to this risk through their hemodynamic effects; evidence from randomized and observational studies has shown reduced left ventricular (LV) mass following AVF ligation [5, 6], but its effect on cardiovascular outcomes remains uncertain.

Clinical decisions regarding VA management are influenced by factors that are not yet fully defined, including the risk of future complications and the feasibility of reconstructing ligated AVFs for future HD access. This underscores the need for a multidisciplinary approach, where patient involvement and informed decision-making are crucial. Yet, surveillance and routine VA evaluations after KT are underutilized, with only 29% of physicians performing such assessments [7]. Moreover, no international guidelines currently address VA management in KT recipients.

This position paper, developed by the Vascular Access Society (VAS) and the European Kidney Transplant Association (EKITA) Section of the European Society of Transplantation (ESOT), reviews current evidence and provides a structured algorithm to support VA monitoring and individualized decision-making in KT patients.

## Current Management of Vascular Access After Kidney Transplantation

There is no consensus on managing VA in asymptomatic patients after kidney transplant (KT). Generally, nephrologists monitor VA and refer to specialists if complications arise. A multinational survey revealed no consensus about ligation of AVF after KT, and most centers do not have a defined protocol for management of AVF after KT [7]. Data on the current practice of VA management after KT and guideline recommendations are scarce. Post-transplant VA ligation is rare, occurring in 4.6% of patients according to the United States Data System [8]. The rate varies among transplant centers: 11% of centers performed ligation on over 10% of KT recipients within a year, while 43% did not perform any ligations among 248 centers. Ligation is typically for patients with steal syndrome or complications like infections and aneurysms. A significant association exists between longer durations on HD (up to 5 years) and AV access ligation after adjusting for donor factors [8]. Longer patency times may increase complications and the need for VA ligation. US data also shows that KT AV access ligation does not affect kidney graft outcomes or reduce all-cause mortality.

The type and placement of VA are also important. Synthetic grafts typically thrombose spontaneously within the first year after KT. Upper-arm AVFs are more likely to require ligation due to local problems than forearm AVFs [9]. The brachiocephalic fistula typically results in higher cardiac output than forearm AVF, leading to a greater incidence of steal syndrome and aneurysm formation [10]. Cephalic arch stenosis can cause giant aneurysms and perforation over time. The frequent stenosis site is a proximal swing segment for patients with basilic vein transposition, which can again cause aneurysmatic complications [11, 12]. Therefore, patients with proximally located AVFs should be evaluated more carefully for ligation or flow reduction.

## Impact of Arteriovenous Access on the Heart and Circulation

The presence of AVFs and grafts significantly influences the CV system. These effects can be categorized based on timing: early or acute changes, which occur immediately after creation, and chronic or delayed changes, which develop over weeks or months (Table 1, adapted and modified from Basile and Lemonte [13]).

**TABLE 1:** Early and late effects of the AVF on the heart and circulation.

Acute effects [days]	Chronic effects [weeks and months]
↓ Systemic vascular resistance	↑ Left ventricular end-diastolic volume
↑ Heart rate, ↑stroke volume	↑ Left ventricular mass and size
↑ Cardiac output	↑ Atrial chamber size
↑ ANP and BNP	Diastolic and systolic dysfunction
↑ Pulmonary flow and pressure	Pulmonary hypertension

Adopted from Basile and Lomonte [13].

## Acute Cardiac Effects of AVF Creation

**FIGURE 1:**
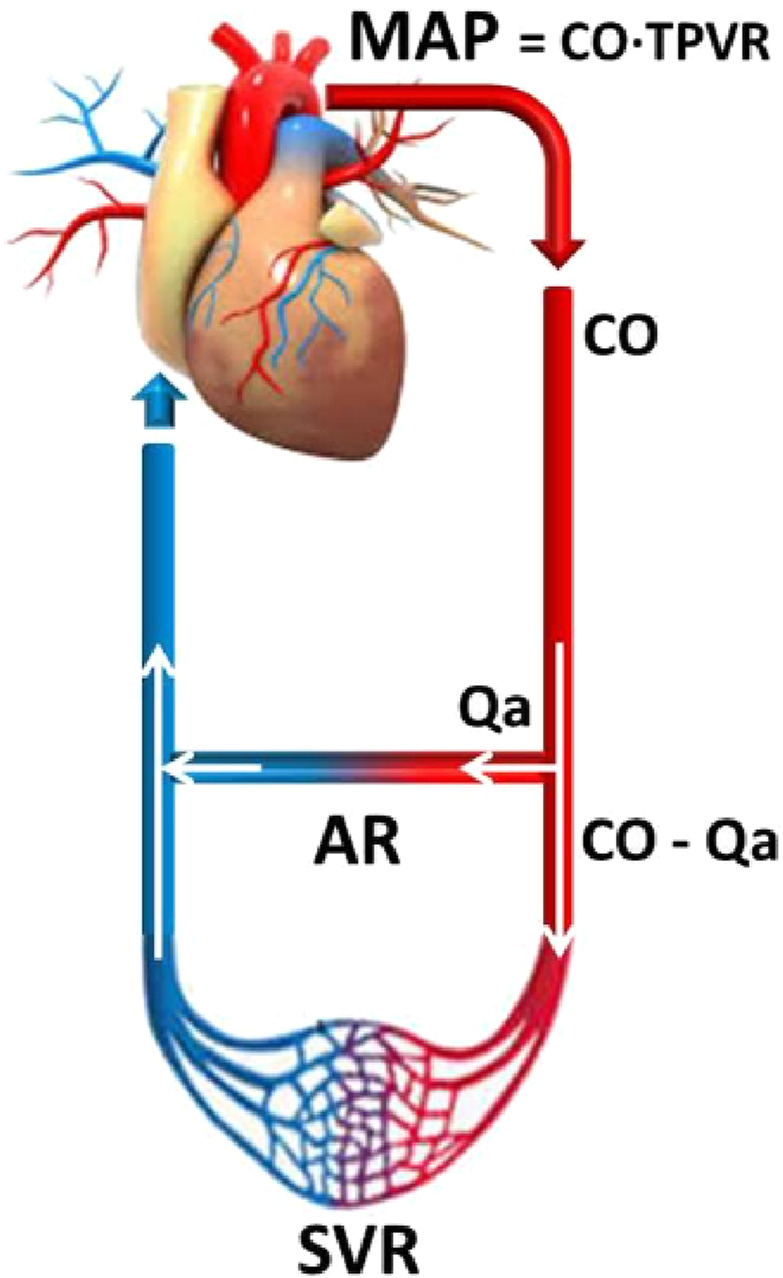
As blood is pumped out of the left ventricle into the arteries, pressure is generated. MAP is determined by CO, TPVR and CVP, according to the following relationship, which is based upon the relationship between flow, pressure and resistance [14]: MAP = (CO TPVR) + CVP. Because CVP is usually at or near 0 mmHg, this relationship is often simplified to MAP = CO TPVR. AR and SVR are connected in parallel Basile C, et al. [13].

Creating an AVF induces an immediate decrease in peripheral vascular resistance, significantly increasing blood flow through the newly created AVF (Figure 1 Basile et al. [15]). Increased blood velocity raises the tangential pressure on the arterial wall, known as wall shear stress (WSS). WSS stimulates endothelial cells to produce vasodilatory substances like nitric oxide (NO), leading to an expansion in the vessel diameter, a reduction in systemic pressure, and the desired decrease of WSS [16]. This new hemodynamic condition places greater demands on the heart; i.e., the heart should increase its work to maintain the blood pressure and equalize the inflow with the outflow to distant organs [17]. As heart rate and stroke volume increase, so does cardiac output (CO) [the amount of blood pumped by the heart in liters per minute]. The increased stretching of cardiac myofibrils also results in elevated production of natriuretic peptides [ANP and BNP] [18]. The diameter of the feeding artery directly influences the newly created conditions of a hyperdynamic circulation. According to Poiseuille’s law, a larger artery diameter will cause a higher flow through the AVF [19]. Likewise, the amount of AVF flow [Qa] and the functional condition of the myocardium influence the type and extent of changes that will manifest over time. In addition to the artery’s diameter, the flow through the AVF also depends on the size of the anastomosis. Flow may decrease due to the presence and onset of stenosis, but it may also gradually increase over months and years of presence. Aneurysm formation in arteriovenous conduit is more common among younger patients with native AVF due to arterial dilatation and anastomotic remodeling [20, 21].

## Chronic Cardiac Effects of Arteriovenous Conduits

Once created and matured, the AVF continuously affects the heart and circulation. Although the adverse effect of the AVF on the heart depends on the AVF flow (Qa), it also depends on the heart itself, as the heart is often affected by several structural and functional alterations in patients with chronic kidney disease (CKD). Therefore, no universal definition of a “safe” Qa exists, but values >1,500–2000 mL/min are usually considered as high and potentially detrimental to the heart [16, 22]. The effects of high Qa act in concert with water overload and include dilatation of all chambers, secondary valvular regurgitation, left ventricular hypertrophy, diastolic dysfunction, and pulmonary hypertension [23]. While water overload and various metabolic and endocrine changes resolve after a successful KT, the effects of fistula flow persist and may contribute to increased patient morbidity and possibly mortality. Nonetheless, direct evidence for this is still lacking. However, some previous studies revealed an early decline in left ventricular mass index (LVMI) and LV end-diastolic diameter (LVEDD) following AVF ligation [23, 24]. Rao et al. conducted a randomized controlled trial (RCT) involving two groups of patients 12 months post-kidney transplantation [5]. Both groups had initial cardiac MRI. One group underwent arteriovenous fistula (AVF) ligation, while the other was a control. A follow-up cardiac MRI 6 months later revealed a noteworthy reduction in left ventricular mass of 22.1g (95% confidence interval: 15.0–29.1) in the AVF ligation group. In comparison, the control group experienced a slight increase of 1.2 g (95% CI: −4.8–7.2) (P < 0.001). This result highlights the potential benefits of AVF ligation in improving cardiac health in kidney transplant recipients. Furthermore, another prospective RCT conducted by Hetz et al. showed that performing prophylactic ligation of a high-flow arteriovenous fistula (AVF) with a flow rate greater than 1,500 mL/min in asymptomatic KT recipients with stable renal function led to a reduction in both proBNP levels and systolic pulmonary arterial pressure (PAP) values [14]. Notably, none of the patients in the group that underwent AVF ligation developed high-output heart failure (HOHF). In contrast, among the patients who did not receive AVF ligation, 5 out of 13 (38.5%) developed HOHF (p < 0.013), indicating that *de novo* heart failure in KT recipients was more frequent in patients with the presence of a functioning AVF (adjusted hazard ratio 2.14). A recent 10-year observational cohort study of 1,330 kidney transplant patients found that those with AVF had a higher incidence of *de novo* heart failure (HF), at 58 cases per 1,000 person-years (95% CI 50–67), compared to 33 cases per 1,000 person-years (95% CI 27–41) in those without AVF, meaning that *de novo* HF was associated with the presence of an AVF [adjusted hazard ratio (aHR) 2.14 (95% CI 1.40–3.26), Moreover, the presence of an AVF was also associated with the composite CV outcome [aHR 1.91 (95% CI 1.31–2.78) [25]. Clinical presentation of the heart changes in CKD patients includes heart failure [HF] of any phenotype [with reduced, mildly reduced, or preserved ejection fraction or high-output HF] and pulmonary hypertension [15]. The progression of HF often leads to a gradual decrease in CO and to its so-called normalization of the CO to a value without the presence of an AVF, which inevitably leads to reduced blood supply to peripheral organs. This situation is called “systemic steal” and results in hypoperfusion of various organs [26]. A recent meta-analysis by Yasir et al., which included over 18,000 transplant patients, adds further evidence to previous studies. It confirms that ligation of symptomatic AVFs in high-output heart failure patients is safe and effective. Additionally, the review identified an AVF flow to cardiac output ratio greater than 0.3 as a predictive marker for the risk of acute heart failure [27].

## Immunosuppression and Vascular Access-Related Aneurysmal Complications

Studies suggest immunosuppressive medication may promote arterial and venous remodeling. [28–30]. Viscardi et al. reported larger AVF venous aneurysms with intense T-lymphocytic infiltrate in patients on immunosuppressive therapy [30], which are prone to thrombosis and significant thrombophlebitis requiring surgery [31]. Brachial artery aneurysm (dilatation of brachial artery >10 mm or more than 50% increase in longitudinal diameter) can be frequent and was detected in 21% of transplant patients [32]. Brachial artery aneurysm can cause ischemia of the arm and frequently requires major vascular surgery for arterial repair. AVF flow volume of more than 1,500 mL/min is associated with a 4.5-fold risk of brachial artery aneurysm formation [32]. The high flow causes upregulation of the local production of vasodilator agents and matrix metalloproteinases 2 and 9, resulting in loss of vessel wall vasoconstriction. Increased blood flow of AVF causes an increase in wall stress and a decrease in wall thickness. Elastic fiber degeneration and increased calcium and phosphate deposition can also affect the long-term [21, 33]. Ligation or flow reduction of the VA augments or prevents the increase of the brachial artery size [34]. Various case reports have shown that aneurysmal degeneration of the inflow artery is a potential serious complication and is mainly associated with immunosuppression [35–38]. These complications predisposed by immunosuppression support a strategy to close AVFs in KT patients with high blood flow, large aneurysms, and good kidney allograft function.

Malignancies confined to AVFs are rare but have been described in case series and reports. They present as angiosarcoma [39–41] and post-transplant lymphoproliferative disorder [42], confined to AVFs mostly in immunosuppressed patients. The most common presenting symptom was pain, with or without a mass. A comprehensive review revealed that of 22 unique patient cases, 19 were post-transplant, and 18 were on antirejection agents [43].

## Surgical Techniques for the Intervention of Arteriovenous Fistulas and Grafts

Revisions for reducing AVF blood flow post-KT include distal inflow, plication, or banding. Short interpositions with small-diameter prosthetic grafts show 58% primary and 71% secondary patency over 3 years [44], while distal inflow revisions demonstrate 48% and 84% [45]. AVF aneurysmography is associated with improved patency and decreased VA abandonment compared to interposition grafting at 2-year follow-up [46]. Some techniques use real-time flow measurements for precise adjustment during surgery [47, 48]. Possible complications include reoperation to reduce blood flow, early and late thrombosis, and infection. Long-term outcomes for transplant patients remain unpredictable, especially with an estimated graft survival of over 10 years.

Therefore, AVF ligation is almost certainly the most viable surgical option. The main risk of ligation, particularly for large AVFs, is that the massive reduction in blood flow in the draining AVF vein leads to thrombosis and the development of thrombophlebitis, which may cause significant discomfort. Therefore, in addition to disconnection of the AVF near the original arteriovenous anastomosis, excising particularly large or aneurysmal segments of the draining vein may be necessary. For this reason, timely management can prevent the loss of venous capital and prohibit the use of draining venous segments [forearm cephalic or upper arm cephalic vein] for future AVF creation. The complication rate of ligation of the VA is relatively limited, as approximately 5% of patients experience post-operative complications, including hematoma and wound infections [49].

## Ligation of VA and Reconstruction in the Future for Hemodialysis: Switch off and on

Considering the cardiac burden and the VA-related complications after KT, the ideal option would be to pause the patency of VA and reintroduce it at the time of switch to HD. Whether thrombosed from a juxta-anastomotic occlusion or surgically ligated right at the anastomosis, reconstructing an occluded AVF is possible for kidney recipients returning to dialysis, even years after occlusion. Forearm AVFs often have early anastomotic stenosis, but matured venous conduits enable successful reconstruction in most cases. Weyde et al. reported that 85 out of 112 forearm AVFs were successfully reconstructed with a one-year primary patency rate of 57.6% [50]. Another series presented the creation of a new VA after kidney failure for patients with an occluded AVF at the distal part of the dominant [87%] or non-dominant [29%] extremity [31]. Other series also present the reconstruction and immediate cannulation of the ligated/thrombosed AV fistulas at the time of switch to HD [51, 52]. The perioperative complication of ligating an uncomplicated forearm AV appeared very low [53, 54]. Reconstructing brachiocephalic AVFs at HD initiation post-KT is possible but uncommon.

Reconstructing ligated or thrombosed AVFs at HD resumption would reduce the need for CVC placement, as the venous conduit can often be cannulated immediately. Forearm AVFs can thrombose up to the antecubital fossa, but if 10–15 cm of venous conduit remains, reconstruction of inflow and outflow is feasible with thrombectomy even years later. Notably, many transplant patients with lower arm AVFs have opportunities for ligation and reconstruction. VA specialists should evaluate this option to enable more informed decision-making involving patients.

## Patient Perspective

Individualized care for CKD patients and access to predialysis information are crucial in nephrology. Current CKD guidelines underline the patient-centered care, with the proper access, at the right time, for the right patient and reason [55]. The European Renal Best Practice remarks that patients prioritized adverse effects of AV accesses and involvement in care, while clinicians focused on options and technical aspects like maturation and patency [56]. Living with a buzzing AVF can cause discomforting psychological and aesthetic influence on KT patients. Among KT recipients, 23% considered ligation (2/3 for esthetics, 1/3 for heart health), 39% opposed it, and 39% had no opinion [57]. In this regard, the information from the vascular access specialist about the possibility of ligating AVF and reconstructing in the future at the time of switch to HD can have an important impact on the patient’s decision for VA ligation. Some studies highlight the importance of an individualized approach to the VA after a successful kidney transplant [58].

## Impact of Arteriovenous Access on the Kidney Allograft

In CKD patients, creating an AVF has been linked to a slower decline in GFR. Golper et al. found that GFR decline dropped from −5.9 to −0.5 mL/min/year after AVF creation in 123 patients (P < 0.001), though without a control group [9]. A larger nationwide cohort of 3026 US patients in 2017 revealed GFR decline slowing from −5.6 to −4.1 mL/min/year post-AVF surgery (P < 0.001) [59]. Hahn Lundstrom compared GFR decline between 435 patients with VA and 309 patients with peritoneal catheter, finding both groups benefited similarly, revealing the benefit of multidisciplinary follow-up [60]. Recently, a Canadian study including a propensity-score matched cohort of future peritoneal dialysis patients without access surgery concluded that the VA placement increased the patients’ awareness of their CKD condition [61]. The last two studies suggest that the key is multidisciplinary follow-up rather than hemodynamic changes.

Theories behind reduced GFR decline include ischemic preconditioning, arterial blood pressure control, and increased venous return to the lungs [62]. Ischemic preconditioning releases erythropoietin, nitric oxide, and adenosine into circulation, protecting organs from ischemic injuries [62–64]. AVF creation significantly lowers central systolic [-8%] and diastolic [-9%] blood pressure [65]. Increased venous return to the lungs boosts oxygen delivery to peripheral organs, including the kidney parenchyma [62, 66]. Considering the adverse long-term effects of hypertension on renal function, AVF ligation and the resulting rise in systemic blood pressure might negatively impact renal allograft function in KT recipients [67, 68].

The first study at KT was published in 2010 by Vajdič and coworkers, who compared kidney graft function and survival between patients with a functioning AVF 1 year after transplantation with patients with a non-functional AVF [69]. The total population included 311 patients, with a mean age of 47 ± 11 years. In a crude analysis, patients with a functioning AVF had worse renal function at 1 year [69 ± 21 mL/min/1.73 m^2^] than those with non-functional AVF [74 ± 19 mL/min mL/min/1.73 m^2^, P < 0.05]. Also, the 5-year graft survival was higher in patients with a non-functioning AVF [75%] than in those with a functioning one [60%]. In a more recent paper published in 2017, Weekers et al analyzed the impact of AVF ligation in a retrospective cohort of 285 kidney transplant recipients with a mean age of 50.2 ± 14.3 years divided into three groups: (no AVF, closed AVF, and left-open AVF). The lowest GFR slope was evident in patients who had their AVF closed [-0.081 mL/min/month] in comparison with the other two groups [-0.183 mL/min/month for patients without AVF and −0.164 with patients with left-open AVF] [70]. In line with this observation, no significant effect of access ligation on GFR was observed in the randomized clinical trial on the impact of access ligation on left ventricular hypertrophy [5].

The conflicting data from these studies do not provide clear-cut information replicating the findings observed in the general population with CKD. Ideally, a large multinational clinical trial in which patients are randomized for closure or left-open AVF after transplantation would provide the best option to prevent renal function deterioration. However, considering the potential benefit of AVF ligation on CV structure [6], this putative trial should also focus on CV events and patient survival.

## Probability of Still Having a Functional AVF at the Time of Allograft Failure

The natural progression of VA following KT was described in a retrospective cohort study of 626 patients. The study reported AVF patency rates of 82%, 70%, and 61% at 1, 3, and 5 years, respectively [31]. Their conservative approach included AVF examination at each clinical visit and ligation only in severely problematic cases. AVF ligation was performed in 24% of patients. Of 127 patients, 53 (40.1%) restarted HD with their original pre-transplant AVF, 12 (9.1%) with a newly constructed AVF, and 7 (9.4%) had the original AVF ligated and reconstructed. The rate of having a functioning AVF at the time of allograft failure in KT patients was 66%, 55% and 14.8% in presented cohorts from Scotland, Italy, and Canada [71–73], illustrating that local preferences and practices have a significant impact on VA management and outcomes. The Canadian study revealed that the 12-month predialysis and 24-month postdialysis VA creation rate was 16% and 47%, respectively [73]. Reports from the US show that nearly two-thirds of patients restart HD with catheters [2]. Therefore, transplantation nephrologists may be so focused on saving the kidney graft that they can postpone management to create AVF when switching to HD [74, 75].

## The Pros and Cons of Ligating a Functional Vascular Access After Transplantation

As mentioned previously, solid data regarding the long-term clinical effects of a functional VA after KT are scarce, and no international guidelines on the management of kidney transplant recipients provide guidance on post-transplant VA management [55, 56, 67, 76–78]. A multicenter survey showed that disagreement among experts among respondents was considerable regarding the decision to ligate AVF after KT, as in four out of eight cases, less than 70% of respondents agreed on the arteriovenous fistula management strategy [7]. Having a functional VA may facilitate rapid access to HD treatment in patients experiencing post-transplant complications, such as delayed graft function or allograft failure, avoiding the need for placing a CVC, which causes an increased risk of infection [79, 80]. Moreover, having an AVF may permit a straightforward initiation of pharmacological or apheresis treatments in patients with poor quality peripheral veins or VA problems developing immune-mediated post-transplant complications, such as episodes of antibody-mediated rejection and recurrence of glomerulonephritis [81]. Belatecept maintenance treatment through AVFs is a good example of IV treatment use of AVFs after KT, which prevents the risk of complications introduced by a port catheter.

In kidney transplant recipients with stable allograft function, ligation of the AVF may reverse maladaptive heart alterations such as right ventricular [RV] dilatation [82], AVF-associated volume overload leads to LV hypertrophy and cardiac remodeling [83], and diastolic dysfunction with structural heart disease [82–84]. Although unproven, this may mitigate the adverse CV clinical effects associated with a long-lasting AVF, such as LV hypertrophy [85], contributing to the increased risk of CV mortality observed among KT patients [86, 87].

Ligation may also reduce the risk of high-output cardiac failure secondary to high-flow AVF [23]. Unger et al. reported that AVF surgical ligation reduced LV end-diastolic diameter and mass indexes 2 months after surgery in stable KT patients. However, diastolic and mean arterial BP were marginally augmented in these patients after AVF ligation [88]. One randomized clinical trial on the cardiac effects of VA ligations has been published [5]. This Australian study included 63 adult patients who underwent successful KT at least 12 months prior to the intervention. MRI assessed cardiac dimensions at baseline and 6 months later. The primary outcome was LV mass reduction at 6 months, which decreased by 22.1 g in the group with AVF ligation and increased by 1.2 g in the control group [p < 0.001]. The cardiac output decreased from 6.8 L/min at baseline to 4.8 L/min at 6 months [p < 0.05] upon AVF ligation. The closure group also observed significant decreases in LV end-diastolic volumes, LV end-systolic volumes, atrial volumes, and NT pro-BNP. Subsequent follow-ups of the patients who underwent AVF ligation revealed a further reduction in LV mass 5 years after AVF ligation [6]. This trial confirms that ligation of AVF in stable post-transplant patients improves LV remodeling.

Late AVF complications in patients following KT are quite common [31, 32]. Preventing the increase in the size and the result of increasing blood flow from the brachial artery is important. Surgical intervention can cease or decrease the diameter of the brachial artery [34]. Therefore, increased blood flow through the AVF, brachial artery aneurysm, and following complications, including venous aneurysm formation, can be prevented by timely ligation or flow-reducing surgery of the VA.

## Proposed Protocol for Management of Vascular Access After Transplantation

**FIGURE 2:**
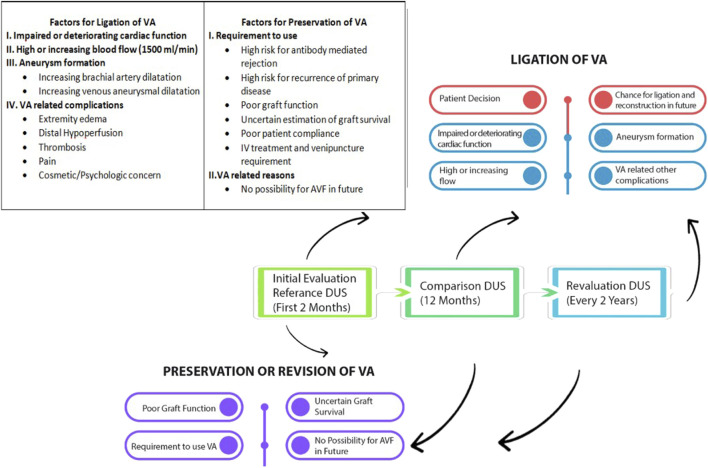
Graphic illustration of the proposed follow-up and management of arteriovenous vascular access after KT.

Effective VA management after KT involves a surveillance protocol. Our proposed protocol is depicted in Figure 2. Initially, the complete VA-related medical history should be documented. Use Doppler ultrasonography to screen VA in the first 2 months post-KT, assessing blood flow, brachial artery dilatation, venous conduit dilatation, and aneurysms. If the duplex ultrasonography reveals a blood flow ≥1 L/min, cardiac evaluation is required to estimate VA-related cardiac morbidity, also considering other comorbidities such as diabetes mellitus and hypertension. Echocardiography should establish baseline cardiac parameters for comparison with follow-up measurements at the end of the first year post-KT. Follow AVG conservatively unless there are cardiac complications, as they usually thrombose spontaneously. Monitor atypical VA conduits and upper arm VAs more carefully due to their risk of aneurysms. Refer VAs with cardiac or access-related complications to specialists immediately. Consider ligation of non-complicated VAs by the end of the first year post-KT.

Evaluating the ligation of VA 1 year post-KT is crucial for several reasons. First, it allows assessment of kidney graft survival, ensuring preservation of VA in recipients with poor outcomes. A banding operation can reduce VA blood flow to preserve it for recipients requiring VA after KT. Second, most of the spontaneous thrombosis of the VA occurs during the first year after KT. Finally, a one-year follow-up helps monitor changes in VA blood flow, aneurysm progression, and their impact on cardiac health.

The evaluation of VA in the initial year after KT should include Doppler ultrasound and examination by a VA specialist. The VA specialist should identify the VA likely to cause future complications. The VA with increasing blood flow, causing increasing brachial artery dilatation, and aneurysm size of venous conduit compared to the initial duplex Ultrasound evaluation should be referred for ligation if the estimated graft survival is over 10 years. Determining if the VA can be reconstructed after ligation is crucial for transplant failure cases, particularly with non-complicated forearm AVFs. A multidisciplinary team, including a nephrologist, cardiologist, and VA specialist, should decide on preservation and ligation in consultation with the patient. In case of preserving the VA, the evaluation should be repeated every 2 years or earlier in case of cardiac complications or VA-related problems, especially when the blood flow of the VA is higher than 1.5 L/min.

## Conclusion

The VA of KT transplant recipients is a modifiable factor that could significantly impact their burden of cardiac disease. Recent studies have demonstrated that ligation of VA results in a substantial and permanent reduction of cardiac hypertrophy. Prospective studies are necessary to evaluate whether AVF closure in asymptomatic patients offers benefits for CV mortality.

High-flow VAs can also cause arterial and venous aneurysmal complications in KT patients, which can be enhanced using immunosuppressive medication. Therefore, implementing a VA surveillance program is essential for improving VA management post-KT. Detecting the subset of transplant patients who may benefit from technical interventions such as ligation and reconstruction of AVFs, particularly when switching back to HD, allows for more informed and patient-involved decision-making regarding VA ligation.

Although the proposed algorithm is not entirely evidence-based, it represents the best management strategy for detecting cardiac and VA morbidity after KT. This approach can contribute valuable data that is currently missing from the literature. Given the favorable prognosis of allografts today, the decision to ligate VA can be considered more liberally, even without VA-related complications, for patients with well-functioning grafts. The decision to maintain or ligate the VA should be made by a multidisciplinary team, with active participation from the patient in the decision-making process.

## References

[1] Poggio ED Augustine JJ Arrigain S Brennan DC Schold JD . Long-Term Kidney Transplant Graft Survival-Making Progress When Most Needed. Am J Transpl (2021) 21(8):2824–32. 10.1111/ajt.16463 33346917

[2] Chan MR Oza-Gajera B Chapla K Djamali AX Muth BL Turk J Initial Vascular Access Type in Patients with a Failed Renal Transplant. Clin J Am Socnephrol (2014) 9(9):1225–31. 10.2215/CJN.12461213 24903392 PMC4078970

[3] Arce CM Lenihan CR Montez-Rath ME Winkelmayer WC . Comparison of Longer-Term Outcomes after Kidney Transplantation between Hispanic and Non-Hispanic Whites in the United States. Am J Transpl (2015) 15:499–507. 10.1111/ajt.13043 25556854

[4] Jardine AG Gaston RS Fellstrom BC Holdaas H . Prevention of Cardiovascular Disease in Adult Recipients of Kidney Transplants. Lancet (2011) 378:1419–27. 10.1016/S0140-6736(11)61334-2 22000138

[5] Rao NN Stokes MB Rajwani A Ullah S Williams K King D Effects of Arteriovenous Fistula Ligation on Cardiac Structure and Function in Kidney Transplant Recipients. Circulation (2019) 139(25):2809–18. 10.1161/CIRCULATIONAHA.118.038505 31045455

[6] Salehi T Montarello NJ Juneja N Stokes MB Scherer DJ Williams KF Long-Term Impact of Arteriovenous Fistula Ligation on Cardiac Structure and Function in Kidney Transplant Recipients: A 5-Year Follow-Up Observational Cohort Study. Kidney360 (2021) 2(7):1141–7. 10.34067/KID.0000692021 35368362 PMC8786094

[7] Voorzaat BM Janmaat CJ Wilschut ED Van Der Bogt KE Dekker FW Rotmans JI . No Consensus on Physicians’ Preferences on Vascular Access Management after Kidney Transplantation: Results of a Multi-National Survey. J Vasc Access (2019) 20(1):52–9. 10.1177/1129729818776905 29843559 PMC6305957

[8] Hicks CW Bae S Pozo ME DiBrito SR Abularrage CJ Segev DL Practice Patterns in Arteriovenous Fistula Ligation Among Kidney Transplant Recipients in the United States Renal Data Systems. J Vasc Surg (2019) 70(3):842–52. 10.1016/j.jvs.2018.11.048 30853386

[9] Golper TA Hartle PM Bian A . Arteriovenous Fistula Creation May Slow Estimated Glomerular Filtration Rate Trajectory. Nephrol Dial Transpl (2015) 30(12):2014–8. 10.1093/ndt/gfv082 25888388 PMC4832989

[10] Van Hoek F Scheltinga MR Kouwenberg I Moret KE Beerenhout CH Tordoir JH . Steal in Hemodialysis Patients Depends on Type of Vascular Access. Eur J Vasc Endovasc Surg (2006) 32(6):710–7. 10.1016/j.ejvs.2006.05.018 16875849

[11] Valenti D Mistry H Stephenson M . A Novel Classification System for Autogenous Arteriovenous Fistula Aneurysms in Renal Access Patients. Vasc Endovascular Surg (2014) 48(7-8):491–6. 10.1177/1538574414561229 25487245

[12] Sivananthan G Menashe L Halin NJ . Cephalic Arch Stenosis in Dialysis Patients: Review of Clinical Relevance, Anatomy, Current Theories on Etiology and Management. J Vasc Access (2014) 15:157–62. 10.5301/jva.5000203 24474522

[13] Basile C Lomonte C . The Complex Relationship Among Arteriovenous Access, Heart, and Circulation. Semin Dial (2018) 31(1):15–20. 10.1111/sdi.12652 28990213

[14] Hetz P Pirklbauer M Müller S Posch L Gummerer M Tiefenthaler M . Prophylactic Ligature of AV Fistula Prevents High Output Heart Failure after Kidney Transplantation. Am J Nephrol (2020) 51(7):511–9. 10.1159/000508957 32659755 PMC7592949

[15] Basile C Vernaglione L Casucci F Libutti P Lisi P Rossi L The Impact of Haemodialysis Arteriovenous Fistula on Haemodynamic Parameters of the Cardiovascular System. Clin Kidney J (2016) 9(5):729–34. 10.1093/ckj/sfw063 27679720 PMC5036899

[16] Malik J Lomonte C Rotmans J Chytilova E Roca-Tey R Kusztal M Hemodialysis Vascular Access Affects Heart Function and Outcomes: Tips for Choosing the Right Access for the Individual Patient. J Vasc Access (2021) 22(1_Suppl. l):32–41. 10.1177/1129729820969314 33143540 PMC8606800

[17] London GM Guerin AP Marchais SJ . Hemodynamic Overload in End-Stage Renal Disease Patients. Semin Dial (1999) 12:77–83. 10.1046/j.1525-139x.1999.00007.x

[18] Iwashima Y Horio T Takami Y Inenaga T Nishikimi T Takishita S Effects of the Creation of Arteriovenous Fistula for Hemodialysis on Cardiac Function and Natriuretic Peptide Levels in CRF. Am J Kidney Dis (2002) 40:974–82. 10.1053/ajkd.2002.36329 12407642

[19] Pfitzner J . Poiseuille and His Law. Anaesthesia (1976) 31:273–5. 10.1111/j.1365-2044.1976.tb11804.x 779509

[20] Sho E Sho M Singh TM Nanjo H Komatsu M Xu C Arterial Enlargement in Response to High Flow Requires Early Expression of Matrix Metalloproteinases to Degrade Extracellular Matrix. Exp Mol Pathol (2002) 73:142–53. 10.1006/exmp.2002.2457 12231217

[21] Tronc F Mallat Z Lehoux S Wassef M Esposito B Tedgui A . Role of Matrix Metalloproteinases in Blood Flow-Induced Arterial Enlargement: Interaction with NO. Arterioscler Thromb Vasc Biol (2000) 20(12):E120–6. 10.1161/01.atv.20.12.e120 11116076

[22] Malik J Valerianova A Tuka V Trachta P Bednarova V Hruskova Z The Effect of High-Flow Arteriovenous Fistulas on Systemic Haemodynamics and Brain Oxygenation. ESC Heart Fail (2021) 8(3):2165–71. 10.1002/ehf2.13305 33755355 PMC8120398

[23] Unger P Wissing KM de Pauw L Neubauer J van de Borne P . Reduction of Left Ventricular Diameter and Mass after Surgical Arteriovenous Fistula Closure in Renal Transplant Recipients. Transplantation (2002) 74(1):73–9. 10.1097/00007890-200207150-00013 12134102

[24] Van Duijnhoven EC Cheriex EC Tordoir JH Kooman JP van Hooff JP . Effect of Closure of the Arteriovenous Fistula on Left Ventricular Dimensions in Renal Transplant Patients. Nephrol Dial Transpl (2001) 16(2):368–72. 10.1093/ndt/16.2.368 11158414

[25] Stoumpos S Van Rhijn P Mangion K Thomson PC Mark PB . Arteriovenous Fistula for Haemodialysis as a Predictor of *De Novo* Heart Failure in Kidney Transplant Recipients. Journal (2024) 17:sfae105. 10.1093/ckj/sfae105 38737344 PMC11087827

[26] Valerianova A Lachmanova J Kovarova L Kmentova T Bartkova M Malik J . Factors Responsible for Cerebral Hypoxia in Hemodialysis Population. Physiol Res (2019) 68:651–8. 10.33549/physiolres.934064 31177793

[27] Yasir MB Man RK Gogikar A Nanda A Niharika Janga LS Sambe HG A Systematic Review Exploring the Impact of Arteriovenous Fistula Ligature on High-Output Heart Failure in Renal Transplant Recipients. Ann Vasc Surg (2024) 100:67–80. 10.1016/j.avsg.2023.10.010 38122973

[28] Mitchell RN Libby P . Vascular Remodeling in Transplant Vasculopathy. Circ Res (2007) 100(7):967–78. 10.1161/01.RES.0000261982.76892.09 17431198

[29] Reilly JM Savage EB Brophy CM Tilson MD . Hydrocortisone Rapidly Induces Aortic Rupture in a Genetically Susceptible Mouse. Arch Surg (1990) 125:707–9. 10.1001/archsurg.1990.01410180025004 2346371

[30] Viscardi A Travaglino A Del Guercio L D’Armiento M Santangelo M Sodo M The Role of Immunosuppressive Therapy in Aneurysmal Degeneration of Hemodialysis Fistulas in Renal Transplant Patients. Ann Vasc Surg (2021) 74:21–8. 10.1016/j.avsg.2021.01.097 33567296

[31] Trampuz BV Arnol M Gubensek J Ponikvar R Ponikvar JB . A National Cohort Study on Hemodialysis Arteriovenous Fistulas after Kidney Transplantation - Long-Term Patency, Use and Complications. BMC Nephrol (2021) 22(1):344. 10.1186/s12882-021-02550-4 34666737 PMC8524975

[32] Janeckova J Bachleda P Utikal P Orsag J . Management of Arteriovenous Fistula after Successful Kidney Transplantation in Long-Term Follow-Up. Transpl Int (2024) 37:12841. 10.3389/ti.2024.12841 39188270 PMC11346416

[33] Dammers R Tordoir JHM Kooman JP Welten RJTJ Hameleers JMM Kitslaar PJEHM The Effect of Flow Changes on the Arterial System Proximal to an Arteriovenous Fistula for Hemodialysis. Ultrasound Med Biol (2005) 31:1327–33. 10.1016/j.ultrasmedbio.2005.03.017 16223635

[34] Janeckova J Bachleda P Koleckova M Utikal P . Brachial Artery Aneurysm as A LATE Complication of Arteriovenous Fistula. J Vasc Access (2023) 24(5):926–32. 10.1177/11297298211059326 34789043

[35] Khalid U Parkinson F Mohiuddin K Davies P Woolgar J . Brachial Artery Aneurysms Following Brachio-Cephalic AV Fistula Ligation. J Vasc Access (2014) 15(1):22–4. 10.5301/jva.5000156 24043327

[36] Mestres G Fontsere N Yugueros X Tarazona M Ortiz I Riambau V . Aneurysmal Degeneration of the Inflow Artery after Arteriovenous Access for Hemodialysis. Eur J Vasc Endovasc Surg (2014) 48(592e596):592–6. 10.1016/j.ejvs.2014.08.011 25224122

[37] Marzelle J Gashi V Nguyen H-D Mouton A Becquemin J-P Bourquelot P . Aneurysmal Degeneration of the Donor Artery after Vascular Access. J Vasc Surg (2012) 55:1052–7. 10.1016/j.jvs.2011.10.112 22322118

[38] Chemla E Nortley M Morsy M . Brachial Artery Aneurysms Associated with Arteriovenous Access for Hemodialysis. Semin Dial (2010) 23:440–4. 10.1111/j.1525-139X.2010.00718.x 20701723

[39] Kittitirapong N Jinawath A Horsirimanont S . Angiosarcoma in Arteriovenous Fistula after Kidney Transplantation. J Vasc Surg Cases Innov Tech (2021) 7(1):142–7. 10.1016/j.jvscit.2020.12.016 33718686 PMC7921173

[40] Paral KM Raca G Krausz T . MYC Amplification in Angiosarcoma Arising from an Arteriovenous Graft Site. Case Rep Pathol (2015) 2015:537297. 10.1155/2015/537297 26682080 PMC4670641

[41] Aldaabil RA Alkhunaizi AM Dawsari NA Dawamneh MF Rabah R . Angiosarcoma at the Site of Nonfunctioning Arteriovenous Fistula in a Kidney Transplant Recipient. J Vasc Surg Cases Innov Tech (2016) 2(2):53–5. 10.1016/j.jvsc.2016.03.004 31193419 PMC6526302

[42] Kousios A Storey R Barnes ET Hamady M Salisbury E Duncan N Plasmacytoma-Like Posttransplant Lymphoproliferative Disease in a Disused Arteriovenous Fistula: The Importance of Histopathology. Kidney Int Rep (2019) 4(5):749–55. 10.1016/j.ekir.2019.02.003 31080933 PMC6506696

[43] Oskrochi Y Razi K Stebbing J Crane J . Angiosarcoma and Dialysis-Related Arteriovenous Fistulae: A Comprehensive Review. Eur J Vasc Endovasc Surg (2016) 51:127–33. 10.1016/j.ejvs.2015.08.016 26482509

[44] Hashimoto T Akagi D Yamamoto S Suhara M Sato O Deguchi J . Short Interposition with a Small-Diameter Prosthetic Graft for Flow Reduction of a High-Flow Arteriovenous Fistula. J Vasc Surg (2021) 73(1):285–90. 10.1016/j.jvs.2020.05.035 32473337

[45] Gerrickens MWM Vaes RHD Govaert B Loon MV Tordoir JH Hoek VH Three Year Patency and Recurrence Rates of Revision Using Distal Inflow with a Venous Interposition Graft for High Flow Brachial Artery Based Arteriovenous Fistula. Eur J Vasc Endovasc Surg (2018) 55(6):874–81. 10.1016/j.ejvs.2018.03.014 29680175

[46] Chang R Alabi O Mahajan A Miller JS Bhat KR Mize BM Arteriovenous Fistula Aneurysmorrhaphy Is Associated with Improved Patency and Decreased Vascular Access Abandonment. J Vasc Surg (2023) 77(3):891–8.e1. 10.1016/j.jvs.2022.10.054 36368647

[47] Lee H Thomas SD Paravastu S Barber T Varcoe RL . Dynamic Banding (DYBAND) Technique for Symptomatic High-Flow Fistulae. Vasc Endovascular Surg (2020) 54(1):5–11. 10.1177/1538574419874934 31506033

[48] Mallios A Gaudin A Haugue A Blic RD Boura B Jennings WC . Customizable Modification of Banding with External Stenting for Arteriovenous Fistula Flow Reduction. J Vasc Surg Cases Innov Tech (2022) 8(2):151–7. 10.1016/j.jvscit.2022.01.003 35330904 PMC8938603

[49] Letachowicz K Banasik M Królicka A Mazanowska O Gołębiowski T Augustyniak-Bartosik H Vascular Access Perspectives in Patients after Kidney Transplantation. Front Surg (2021) 8:640986. 10.3389/fsurg.2021.640986 33996883 PMC8113696

[50] Weyde W Letachowicz W Krajewska M Golebiowski T Letachowicz K Kusztal M Vascular Access Perspectives in Patients after Kidney Transplantation. Clin Transpl (2008) 22:185–90. 10.1111/j.1399-0012.2007.00767.x 18339138

[51] Gołębiowski T Letachowicz K Letachowicz W Kusztal M Garcarek J Strempska B Use of the Subcutaneous Venous Network of the Forearm to Create an Arteriovenous Fistula. Hemodial Int (2015) 19(4):E24–8. 10.1111/hdi.12304 25881485

[52] Ulloa JG Jimenez JC Pantoja JL Farley SM Gelabert HA Rigberg DA Elective Resection of Symptomatic Arteriovenous Fistulae and Grafts in Patients with Functioning Renal Allografts at A High Volume Transplant Hospital. Ann Vasc Surg (2021) 76:449–53. 10.1016/j.avsg.2021.03.048 33905849

[53] Fraser CD Grimm JG Liu RH Wesson RN Azar F . Removal of Non-Infected Arteriovenous Fistulae Following Kidney Transplantation Is a Safe and Beneficial Management Strategy for Unused Dialysis AccessAnn. Vasc Surg (2018) 53:128–32. 10.1016/j.avsg.2018.04.020 29886220

[54] Van der Veer SN Haller MC Pittens CA Broerse J Castledine C Gallieni M Setting Priorities for Optimizing Vascular Access Decision Making--An International Survey of Patients and Clinicians. PloS one (2015) 10(7):e0128228. 10.1371/journal.pone.0128228 26151822 PMC4494812

[55] Lok CE Huber TS Lee T Shenoy S Yevzlin AS Abreo K KDOQI Clinical Practice Guideline for Vascular Access: 2019 Update. Am J Kidney Dis (2020) 75(4 Suppl. 2):S1–S164. 10.1053/j.ajkd.2019.12.001 32778223

[56] Fluck R Kumwenda M . Renal Association Clinical Practice Guideline on Vascular Access for Haemodialysis. Nephron Clin Pract (2011) 118(Suppl. 1):c225–40. 10.1159/000328071 21555898

[57] Bardowska K Letachowicz K Kaminska D Kusztal M Golebiowski T Krolicki T The Attitude of Kidney Transplant Recipients towards Elective Arteriovenous Fistula Ligation. PloS one (2020) 15(7):e0234931. 10.1371/journal.pone.0234931 32615582 PMC7332306

[58] Letachowicz K Bardowska K Królicki T Kamińska D Banasik M Zajdel K The Impact of Location and Patency of the Arteriovenous Fistula on Quality of Life of Kidney Transplant Recipients. Ren Fail (2021) 43(1):113–22. 10.1080/0886022X.2020.1865171 33397180 PMC7801108

[59] Sumida K Molnar MZ Potukuchi PK Thomas F Lu JL Ravel VA Association between Vascular Access Creation and Deceleration of Estimated Glomerular Filtration Rate Decline in Late-Stage Chronic Kidney Disease Patients Transitioning to End-Stage Renal Disease. Nephrol Dial Transpl (2017) 32(8):1330–7. 10.1093/ndt/gfw220 27242372 PMC5837412

[60] Hahn Lundström U Hedin U Gasparini A Caskey FJ Carrero JJ Evans M . Arteriovenous Access Placement and Renal Function Decline. Nephrol Dial Transpl (2021) 36(2):275–80. 10.1093/ndt/gfz221 31665436

[61] Dupuis MÈ Laurin LP Goupil R Bénard V Pichette M Lafrance JP Arteriovenous Fistula Creation and Estimated Glomerular Filtration Rate Decline in Advanced CKD: A Matched Cohort Study. Kidney360 (2020) 2(1):42–9. 10.34067/KID.0005072020 35368820 PMC8785744

[62] Locatelli F Zoccali C . Arteriovenous Fistula as a Nephroprotective Intervention in Advanced CKD: Scientific Discovery and Explanation, and the Evaluation of Interventions. Nephrol Dial Transpl (2015) 30(12):1939–41. 10.1093/ndt/gfv281 26232781 PMC4832992

[63] Bøtker HE Kharbanda R Schmidt MR Bøttcher M Kaltoft AK Terkelsen CJ Remote Ischaemic Conditioning before Hospital Admission, as a Complement to Angioplasty, and Effect on Myocardial Salvage in Patients with Acute Myocardial Infarction: A Randomised Trial. Lancet (2010) 375(9716):727–34. 10.1016/S0140-6736(09)62001-8 20189026

[64] Yang Y Lang XB Zhang P Lv R Wang YF Chen JH . Remote Ischemic Preconditioning for Prevention of Acute Kidney Injury: A Meta-Analysis of Randomized Controlled Trials. Am J Kidney Dis (2014) 64(4):574–83. 10.1053/j.ajkd.2014.04.029 24954246

[65] Korsheed S Eldehni MT John SG Fluck RJ McIntyre CW . Effects of Arteriovenous Fistula Formation on Arterial Stiffness and Cardiovascular Performance and Function. Nephrol Dial Transpl (2011) 26(10):3296–302. 10.1093/ndt/gfq851 21317408

[66] Burchell AE Lobo MD Sulke N Sobotka PA Paton JF . Arteriovenous Anastomosis: Is This the Way to Control Hypertension? Hypertension (2014) 64(1):6–12. 10.1161/HYPERTENSIONAHA.114.02925 24711522

[67] Unger P Wissing KM . Arteriovenous Fistula after Renal Transplantation: Utility, Futility or Threat? Nephrol Dial Transpl (2006) 21(2):254–7. 10.1093/ndt/gfi276 16293634

[68] Vanderweckene P Weekers L Lancellotti P Jouret F . Controversies in the Management of the Haemodialysis-Related Arteriovenous Fistula Following Kidney Transplantation. Clin Kidney J (2018) 11(3):406–12. 10.1093/ckj/sfx113 29992020 PMC6007507

[69] Vajdič B Arnol M Ponikvar R Kandus A Buturović-Ponikvar J . Functional Status of Hemodialysis Arteriovenous Fistula in Kidney Transplant Recipients as a Predictor of Allograft Function and Survival. Transpl Proc (2010) 42(10):4006–9. 10.1016/j.transproceed.2010.09.057 21168612

[70] Weekers L Vanderweckene P Pottel H Castanares-Zapatero D Bonvoisin C Hamoir E The Closure of Arteriovenous Fistula in Kidney Transplant Recipients Is Associated with an Acceleration of Kidney Function Decline. Nephrol Dial Transpl (2017) 32(1):196–200. 10.1093/ndt/gfw351 27798197

[71] Aitken E Kingsmore D . The Fate of the Fistula Following Renal Transplantation. Transpl Int (2014) 27(9):e90–1. 10.1111/tri.12326 24673883

[72] Manca O Pisano GL Carta P Manca EM Piredda GB Pili G The Management of Hemodialysis Arteriovenous Fistulas in Well Functioning Renal Transplanted Patients: Many Doubts, Few Certainties. J Vasc Access (2005) 6(4):182–6. 10.1177/112972980500600405 16552699

[73] Joyce CZ Al-Jaishi A Perl J Garg AX Moist LM . Hemodialysis Arteriovenous Vascular Access Creation after Kidney Transplant Failure. Am J Kidney Dis (2015) 66(4):646–54. 10.1053/j.ajkd.2015.03.031 25975965

[74] Khalil AK Wish JB . Hemodialysis Access in Patients with Failed Kidney Transplants: Nephrologist. Heal Thyself Am J Kidney Dis (2015) 66(4):555–7. 10.1053/j.ajkd.2015.07.003 26408234

[75] Haq NU Abdelsalam MS Althaf MM Khormi AA Harbi HA Alshamsan B Vascular Access Types in Patients Starting Hemodialysis after Failed Kidney Transplant: Does Close Nephrology Follow-Up Matter? J Vasc Access (2017) 18(1):22–5. 10.5301/jva.5000631 27911463

[76] Schmidli J Widmer MK Basile C de Donato G Gallieni M Gibbons CP Editor's Choice - Vascular Access: 2018 Clinical Practice Guidelines of the European Society for Vascular Surgery (ESVS). Eur J Vasc Endovasc Surg (2018) 55(6):757–818. 10.1016/j.ejvs.2018.02.001 29730128

[77] Tordoir J Canaud B Haage P Konner K Basci A Fouque D EBPG on Vascular Access. Nephrol Dial Transpl (2007) 22(Suppl. 2):ii88–117. 10.1093/ndt/gfm021 17507428

[78] Wilmink T Hollingworth L Dasgupta I . Access Ligation in Transplant Patients. J Vasc Access (2016) 17(Suppl. 1):S64–8. 10.5301/jva.5000537 26951908

[79] O'Grady NP Alexander M Burns LA Dellinger EP Garland J Heard SO Healthcare Infection Control Practices Advisory Committee (HICPAC). Guidelines for the Prevention of Intravascular Catheter-Related Infections. Clin Infect Dis (2011) 52(9):e162–93. 10.1093/cid/cir257 21460264 PMC3106269

[80] Bouza E Burillo A Guembe M . Managing Intravascular Catheter-Related Infections in Heart Transplant Patients: How Far Can We Apply IDSA Guidelines for Immunocompromised Patients? Curr Opin Infect Dis (2011) 24(4):302–8. 10.1097/QCO.0b013e328348b1b9 21666455

[81] Karim MS Aryal P Gardezi A Clark DF Aziz F Parajuli S . Vascular Access in Kidney Transplant Recipients. Transpl Rev (Orlando) (2020) 34(3):100544. 10.1016/j.trre.2020.100544 32205010

[82] Xiao Z Rotmans JI . Considering the Closure of Arteriovenous Fistulas in Kidney Transplant Recipients. Kidney360 (2023) 4(8):1019–20. 10.34067/KID.0000000000000235 37651665 PMC10484350

[83] Lee SR Thorn S Guerrera N Gonzalez L Taniguchi R Langford J Arteriovenous Fistula-Induced Cardiac Remodeling Shows Cardioprotective Features in Mice. JVS Vasc Sci (2021) 2:110–28. 10.1016/j.jvssci.2021.05.002 34423320 PMC8375600

[84] Reddy YNV Obokata M Dean PG Melenovsky V Nath KA Borlaug BA . Long-Term Cardiovascular Changes Following Creation of Arteriovenous Fistula in Patients with End Stage Renal Disease. Eur Heart J (2017) 38(24):1913–23. 10.1093/eurheartj/ehx045 28329100

[85] Kolonko A Kujawa-Szewieczek A Szotowska M Kuczera P Chudek J Więcek A . The Association of Long-Functioning Hemodialysis Vascular Access with Prevalence of Left Ventricular Hypertrophy in Kidney Transplant Recipients. Biomed Res Int (2014) 2014:603459. 10.1155/2014/603459 24616896 PMC3925527

[86] Middleton RJ Parfrey PS Foley RN . Left Ventricular Hypertrophy in the Renal Patient. J Am Soc Nephrol (2001) 12(5):1079–84. 10.1681/ASN.V1251079 11316868

[87] Rigatto C Foley R Jeffery J Negrijn C Tribula C Parfrey P . Electrocardiographic Left Ventricular Hypertrophy in Renal Transplant Recipients: Prognostic Value and Impact of Blood Pressure and Anemia. J Am Soc Nephrol (2003) 14(2):462–8. 10.1097/01.asn.0000043141.67989.39 12538748

[88] Unger P Velez-Roa S Wissing KM Hoang AD van de Borne P . Regression of Left Ventricular Hypertrophy after Arteriovenous Fistula Closure in Renal Transplant Recipients: A Long-Term Follow-Up. Am J Transpl (2004) 4(12):2038–44. 10.1046/j.1600-6143.2004.00608.x 15575907

