# Corrigendum: Vascular Access Management After Kidney Transplantation Position Paper on Behalf of the Vascular Access Society and the European Kidney Transplant Association

**DOI:** 10.3389/ti.2025.16128

**Published:** 2026-01-09

**Authors:** Barış Akin, Tamara K. Jemcov, David Cucchiari, Jan Malik, Gavin J. Pettigrew, Ulrika Hahn Lundström, Gianluigi Zaza, Joris I. Rotmans

**Affiliations:** 1 Department of Surgery, Demiroglu Bilim University Florence Nightingale Hospital, Istanbul, Türkiye; 2 European Kidney Transplant Association, Section of European Society of Transplantation, Amsterdam, Netherlands; 3 Department of Nephrology, Clinical Hospital Center Zemun, Belgrade, Serbia; 4 Faculty of Medicine, University of Belgrade, Belgrade, Serbia; 5 Vascular Access Society, Maastricht, Netherlands; 6 Department of Nephrology and Kidney Transplantation, Hospital Clínic of Barcelona, Barcelona, Spain; 7 Complex Cardiovascular Center, General University Hospital, First Medical Faculty, Charles University, Prague, Czechia; 8 Department of Surgery, University of Cambridge, London, United Kingdom; 9 Department of CLINTEC, Division of Renal Medicine, Karolinska Institutet and Karolinska University Hospital, Stockholm, Sweden; 10 Department of Medical and Surgical Sciences, University of Foggia, Foggia, Italy; 11 Department of Internal Medicine, Leiden University Medical Center, Leiden, Netherlands

**Keywords:** ligation of arteriovenous fistula, kidney transplantation, AVF flow reduction, hemodialysis, kidney failure

There was a mistake in Figure 2 as published. The flow rate in the box regarding Factors for Ligation of VA was incorrect (250 mL/min) and should have been (1500 mL/min). The corrected Figure 2 appears below.

**FIGURE 2:**
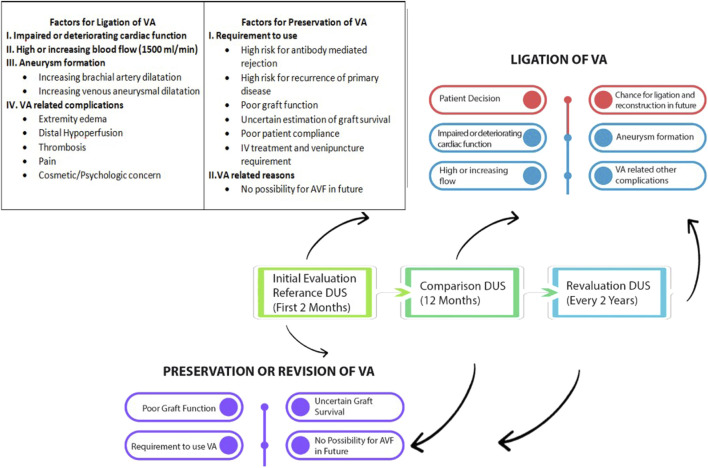
Graphic illustration of the proposed follow-up and management of arteriovenous vascular access after KT.

The authors apologize for this error and state that this does not change the scientific conclusions of the article in any way. The original article has been updated.

